# 34. Long-term clinical outcomes following SARS-CoV-2 infection include persistent symptoms and cardiovascular disease beyond 3 months post-infection

**DOI:** 10.1093/ofid/ofab466.034

**Published:** 2021-12-04

**Authors:** Stephanie A Richard, Simon Pollett, Nusrat J Epsi, Ryan C Maves, Ryan C Maves, Gregory Utz, Tahaniyat Lalani, Rupal Mody, Anuradha Ganesan, Rhonda E Colombo, Chris Colombo, David A Lindholm, David A Lindholm, Cristian Madar, Sharon Chi, Nikhil Huprikar, Derek Larson, Samantha Bazan, Celia Byrne, Caroline E English, Edward Parmelee, Katrin Mende, Mark Simons, Timothy Burgess, David Tribble, Brian Agan

**Affiliations:** 1 Infectious Disease Clinical Research Program, Department of Preventive Medicine and Biostatistics, Uniformed Services University of the Health Sciences, Bethesda, MD and Henry M. Jackson Foundation, Bethesda, MD, Bethesda, Maryland; 2 Uniformed Services University of the Health Sciences, Bethesda, Maryland; 3 HJF, Bethesda, Maryland; 4 Naval Medical Center San Diego, San Diego, CA and Infectious Disease Clinical Research Program, Bethesda, MD, San DIego, California; 5 Naval Medical Center San Diego, Infectious Disease Clinical Research Program, Bethesda, MD, and Henry M. Jackson Foundation for the Advancement of Military Medicine, Inc., Bethesda, MD, San Diego, California; 6 Infectious Disease Clinical Research Program, Bethesda, MD, The Henry M. Jackson Foundation, Bethesda, MD, and Naval Medical Center Portsmouth, VA, Portsmouth, Virginia; 7 WBAMC, El Paso, Texas; 8 Infectious Disease Clinical Research Program and the Henry M. Jackson Foundation for the Advancement of Military Medicine and Walter Reed National Military Medical Center, Bethesda, MD; 9 Madigan Army Medical Center, Tacoma, WA, Infectious Disease Clinical Research Program, Bethesda, MD, and Henry M. Jackson Foundation for the Advancement of Military Medicine, Inc., Bethesda, MD, Tacoma, Washington; 10 Madigan Army Medical Center, Joint Base Lewis-McChord, Washington; 11 Uniformed Services University of the Health Sciences; Brooke Army Medical Center, San Antonio, TX; 12 Tripler Army Medical Center, Tripler Army Medical Center, Hawaii; 13 TAMC, Honolulu, Hawaii; 14 Walter Reed National Military Medical Center (WRNMMC), Bethesda, Maryland; 15 Fort Belvoir Community Hospital Infectious Disease, Fort Belvoir, Virginia; 16 Carl R. Darnall Army Medical Center, Fort Hood, Texas; 17 USUHS, Bethesda, Maryland; 18 Infectious Disease Clinical Research Program, Uniformed Services University of the Health Sciences, Rockville, Maryland; 19 Infectious Disease Clinical Research Program, Bethesda, MD, The Henry M. Jackson Foundation, Bethesda, MD, and Brooke Army Medical Center, Fort Sam Houston, TX, San Antonio, TX; 20 IDCRP, Bethesda, Maryland; 21 Infectious Disease Clinical Research Program, Bethesda, Maryland; 22 Uniformed Services University, Bethesda, MD; 23 Infectious Disease Clinical Research Program, USU/HJF, Bethesda, Maryland

## Abstract

**Background:**

The long-term health effects after SARS-CoV-2 infection remain poorly understood. We evaluated health and healthcare usage after SARS-CoV-2 infection via surveys and longitudinal electronic medical record (EMR) review within the Military Health System (MHS).

**Methods:**

We studied MHS beneficiaries enrolled in the Epidemiology, Immunology, and Clinical Characteristics of Emerging Infectious Diseases with Pandemic Potential (EPICC) cohort from March to December 2020. COVID-19 illness symptom severity and duration were derived from surveys initiated in late 2020. In addition, multi-year healthcare encounter history before and after onset of COVID-19 symptoms was collected from the MHS EMR. Odds of organ-system clinical diagnoses within the 3 months pre- and post-symptom onset were calculated using generalized linear models, controlling for age, sex, and race, and including participant as a random effect.

**Results:**

1,015 participants were included who were SARS-CoV-2 positive, symptomatic, and had 3-month follow-up data available in the EMR (Table 1). 625 of these participants had survey data collected more than 28 days post-symptom onset, among whom 17% and 6% reported persistent symptoms at 28-84 days, and 85+ days, respectively. 9.6% had not resumed normal activities by one month. The most frequently reported symptoms persisting beyond 28 days were dyspnea, loss of smell and/or taste, fatigue, and exercise intolerance (Figure 1A). When compared with the period 61 to 90 days prior to symptom onset, the first month post-symptom onset period was associated with increases of pulmonary (aOR = 57, 95% CI 28-112), renal (aOR = 29, 95% CI 10-84), cardiovascular (aOR = 7, 95% CI 5-11), and neurological diagnoses (aOR = 3, 95% CI 2-4) (Figures 1B and 1C). Cardiovascular disease diagnoses remained elevated through 3 months (aOR = 2, 95% CI 1-3).

Table 1. Characteristics of SARS-CoV-2+ EPICC participants, and illness duration among those with 28+ days post-symptom onset survey data collection.

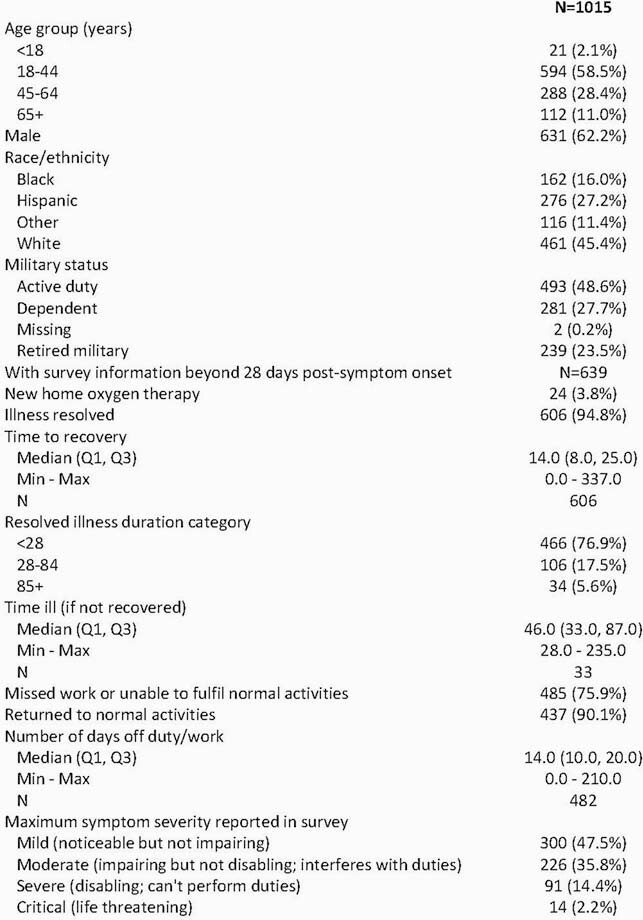

Figure 1

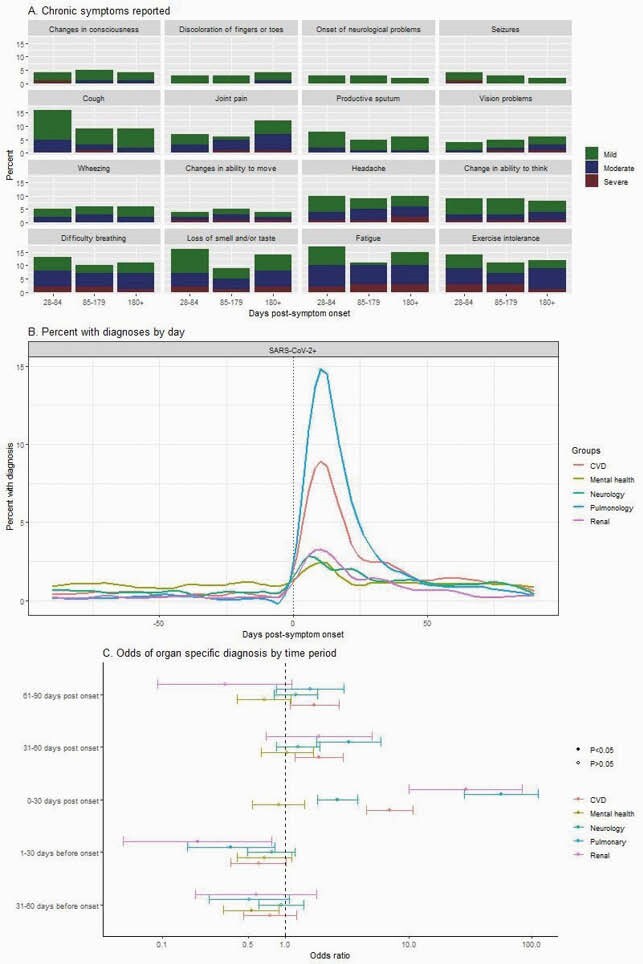

Fig1A. Symptoms reported by EPICC participants with illnesses longer than 28 days; 1B. Percent of participants with organ system specific diagnoses on each day, 90 days pre- and post-symptom onset; 1C. Odds of organ system specific diagnoses within each month, +/- 3 months of symptom onset, were calculated using generalized linear models, controlling for age, sex, and race and included participants as a random effect. Odds shown are relative to the earliest period included in the model, 61-90 days before onset.

**Conclusion:**

In this MHS cohort, a significant proportion of participants had persistent symptoms and cardiovascular disease diagnoses 3 months after COVID-19 illness onset. These findings emphasize the long-term morbidity of COVID-19 and the importance of mitigating SARS-CoV-2 infections. Further analyses will evaluate demographic, clinical, and biomarker predictors of medium-to-long term organ-specific post-acute sequelae.

**Disclosures:**

**Simon Pollett, MBBS**, **Astra Zeneca** (Other Financial or Material Support, HJF, in support of USU IDCRP, funded under a CRADA to augment the conduct of an unrelated Phase III COVID-19 vaccine trial sponsored by AstraZeneca as part of USG response (unrelated work)) **Ryan C. Maves, MD**, **EMD Serono** (Advisor or Review Panel member)**Heron Therapeutics** (Advisor or Review Panel member) **David A. Lindholm, MD**, American Board of Internal Medicine (Individual(s) Involved: Self): Member of Auxiliary R&D Infectious Disease Item-Writer Task Force. No financial support received. No exam questions will be disclosed ., Other Financial or Material Support

